# Utility of CT radiomics for prediction of PD‐L1 expression in advanced lung adenocarcinomas

**DOI:** 10.1111/1759-7714.13352

**Published:** 2020-02-11

**Authors:** Jiyoung Yoon, Young Joo Suh, Kyunghwa Han, Hyoun Cho, Hye‐Jeong Lee, Jin Hur, Byoung Wook Choi

**Affiliations:** ^1^ Department of Radiology Research Institute of Radiological Science, Severance Hospital, Yonsei University College of Medicine Seoul South Korea

**Keywords:** Computed tomography, immunotherapy, lung adenocarcinoma, programmed death ligand 1, radiomics

## Abstract

**Background:**

We aimed to assess if quantitative radiomic features can predict programmed death ligand 1 (PD‐L1) expression in advanced stage lung adenocarcinoma.

**Methods:**

This retrospective study included 153 patients who had advanced stage (>IIIA by TNM classification) lung adenocarcinoma with pretreatment thin section computed tomography (CT) images and PD‐L1 expression test results in their pathology reports. Clinicopathological data were collected from electronic medical records. Visual analysis and radiomic feature extraction of the tumor from pretreatment CT were performed. We constructed two models for multivariate logistic regression analysis (one based on clinical variables, and the other based on a combination of clinical variables and radiomic features), and compared c‐statistics of the receiver operating characteristic curves of each model to identify the model with the higher predictability.

**Results:**

Among 153 patients, 53 patients were classified as PD‐L1 positive and 100 patients as PD‐L1 negative. There was no significant difference in clinical characteristics or imaging findings on visual analysis between the two groups (*P* > 0.05 for all). Rad‐score by radiomic analysis was higher in the PD‐L1 positive group than in the PD‐L1 negative group with a statistical significance (−0.378 ± 1.537 vs. −1.171 ± 0.822, *P* = 0.0008). A prediction model that uses clinical variables and CT radiomic features showed higher performance compared to a prediction model that uses clinical variables only (c‐statistic = 0.646 vs. 0.550, *P* = 0.0299).

**Conclusions:**

Quantitative CT radiomic features can predict PD‐L1 expression in advanced stage lung adenocarcinoma. A prediction model composed of clinical variables and CT radiomic features may facilitate noninvasive assessment of PD‐L1 expression.

**Key points:**

Significant findings of the study

Quantitative CT radiomic features can help predict PD‐L1 expression, whereas none of the qualitative imaging findings is associated with PD‐L1 positivity.

What this study adds

A prediction model composed of clinical variables and CT radiomic features may facilitate noninvasive assessment of PD‐L1 expression.

## Introduction

Lung cancer is the leading cause of cancer‐related deaths worldwide, and adenocarcinoma is the most common histologic type of lung cancer.^1^, ^2^ In the past, platinum‐based conventional chemotherapy was the only option for treating advanced lung adenocarcinoma. However, recent developments in molecular‐targeted therapy has significantly improved survival to subsets of patients who are positive for genetic alteration such as mutation in the epidermal growth factor receptor (*EGFR*) gene and rearrangement of the anaplastic lymphoma kinase gene locus.^3^, ^4^

Recently, immune checkpoint inhibitors targeting programmed cell death protein 1 (PD‐1) or programmed death ligand 1 (PD‐L1) have demonstrated better progression‐free and overall survival than conventional chemotherapy in advanced non‐small cell lung cancer (NSCLC) patients.^5^, ^6^, ^7^ As immunotherapy became one of the standard treatment regimens for NSCLC, biomarkers for predicting responses to immune checkpoint inhibitors were investigated and PD‐L1 expression on tumor cells was accepted as a predictive biomarker for the immunotherapy response.^8^, ^9^, ^10^ In this context, the International Association for the Study of Lung Cancer (IASLC) provided an atlas of PD‐L1 immunohistochemistry testing in NSCLC.^11^

The prediction of PD‐L1 expression from computed tomography (CT) imaging features may have value, not only for predicting patient outcome by imaging, but also in situations where tissue sampling is not possible. Previous studies have investigated the relationship between CT image features and PD‐L1 expression.^12^, ^13^ However, these studies focused primarily on qualitative imaging features with study populations that were limited to early stage, resectable lung adenocarcinomas; therefore, quantitative analysis may be more valuable. “Radiomics,” an emerging tool that provides quantitative imaging parameters, has been applied in oncology for tumor assessment and evaluation of the patient's response to treatment (e.g. prediction of *EGFR* mutation and response to the targeted therapy in NSCLC).^14^, ^15^, ^16^, ^17^, ^18^, ^19^ Because a radiomics approach can provide objective and quantitative parameters of the tumor, we hypothesized that quantitative radiomic features can predict PD‐L1 expression in advanced stage lung adenocarcinoma.

Therefore, the purpose of this study was to assess if quantitative radiomic features can predict PD‐L1 expression in advanced stage lung adenocarcinoma.

## Methods

### Patients

**Figure 1:**
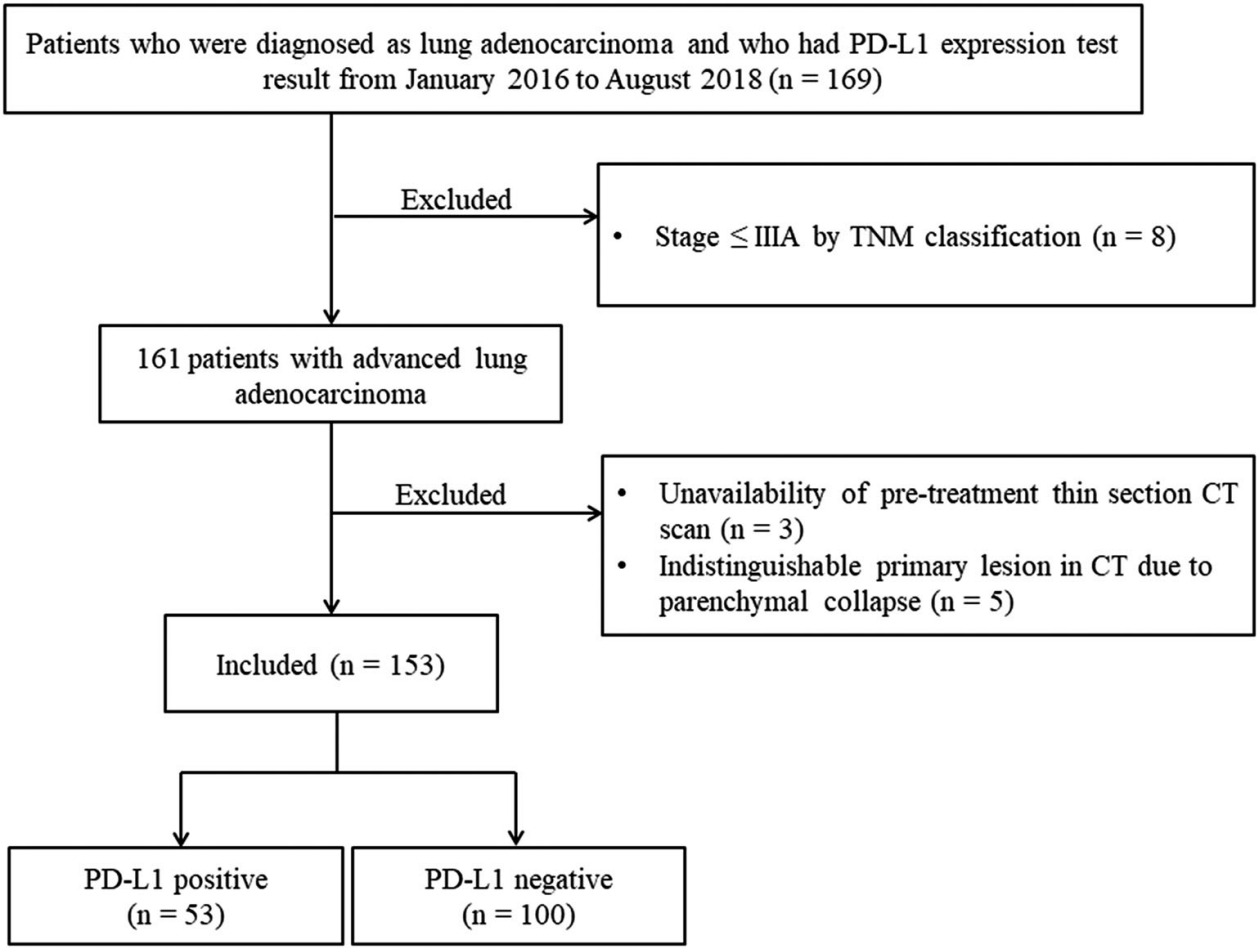
Patient selection diagram. CT, computed tomography; PD‐L1, programmed death ligand 1.

Our institutional review board approved this retrospective study, and the requirement for obtaining informed consent was waived. We conducted a retrospective chart review, and identified 169 patients who were diagnosed with lung adenocarcinomas from January 2016 to August 2018 and whose pathological reports included a PD‐L1 expression test result obtained by tumor proportion score (TPS). Among these 169 patients, 16 patients were excluded from this study for the following reasons: (i) a resectable stage of NSCLC (≤stage IIIA by TNM classification according to the eighth edition of IASLC)^20^ (*n* = 8); (ii) unavailability of thin section CT images prior to treatment (*n* = 3); and (iii) indistinguishable primary lesion in CT scan due to parenchymal collapse (*n* = 5). A total of 153 patients were included in the study who were diagnosed in pathological reports as having advanced stage lung adenocarcinoma and having a PD‐L1 expression test result obtained by TPS (99 men, mean age 64.6 ± 10.7 years, range, 34–86 years) (Fig 1).

Clinicopathological data collected for each patient included age, gender, smoking history, TNM stage, PD‐L1 expression status by TPS, and *EGFR* mutation status.

### Chest computed tomography (CT) examinations

For all patients, contrast‐enhanced chest CT scans were performed by using one of following multidetector row scanners: Somatom Sensation 16, Somatom Sensation 64, Definition Flash (Siemens Medical Solutions, Forchheim, Germany), Discovery CT 750 HD, Revolution (GE Medical Systems, Milwaukee, Wisconsin, USA), or iCT (Philips Medical Systems, the Netherlands). Details of scanning parameters were the same as previously described.^21^ A bolus of 50–90 mL (1.5 mL/kg bodyweight) of iopamidol (300 mg I/mL, Radisense, Taejoon Pharmaceutical, Seoul, South Korea) was injected intravenously at a flow rate of 3 mL/second for enhanced images, and an automated bolus‐tracking technique was used. Axial and coronal images were reconstructed with soft tissue kernel and a slice thickness of 1–1.25 mm and 2.5–3 mm, respectively. All CT datasets were transferred to a picture archiving and communication system.

### Visual analysis of CT images

Visual analysis was performed by two board‐certified thoracic radiologists (with nine and 10 years' experience in chest CT imaging, respectively) who were blinded to the clinical and histologic findings. Two radiologists independently reviewed all CT images, and any discrepancies in evaluations were resolved by agreement. CT images were read on the axial and coronal views with both mediastinal (width, 350 HU; level, 40 HU) and lung (width, 1500 HU; level, −500 HU) window settings. CT image features that were included in the visual analysis were as follows^22^, ^23^: (i) size (maximal and minimal diameters), location, type (nodule, mass, multicentric, or ground‐glass opacity [GGO]/consolidation), and margin (lobulation, concavity, spiculation) of primary mass; (ii) internal characteristics of tumor: presence of internal calcification, air bronchogram, bubble‐like lucency, cavitation, or necrosis; (iii) external characteristics of tumor: fissural or pleural attachment, thickening of adjacent bronchovascular bundles, pleural retraction, or peripheral emphysema; and (iv) associated findings: pattern of lung metastasis, presence of pleural effusion, pleural nodularity, significant pericardial effusion (moderate to large amount [>10 mm in depth] or pericardial nodularity or enhancement regardless of size), intrathoracic bony metastases, or metastatic lymphadenopathy.

### CT radiomic feature extraction

**Figure 2:**
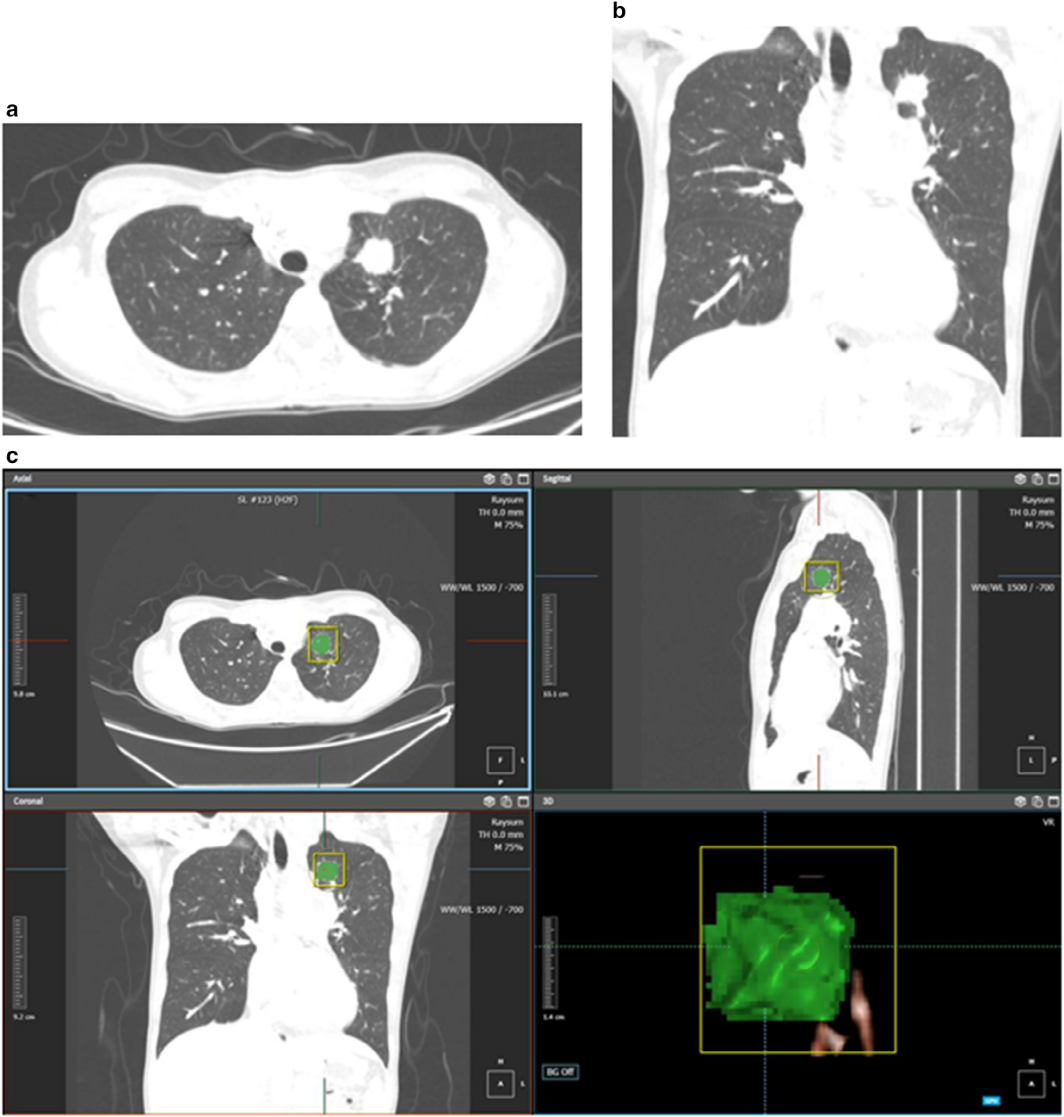
CT images and radiomic analysis of a 34‐year‐old female with adenocarcinoma positive for PD‐L1 expression. Axial (a) and coronal (b) images of the initial contrast‐enhanced CT scan show a 2.3 cm nodule in the left upper lobe which appears as a pure solid nodule with spiculation and pleural attachment. Multiple metastatic left hilar, bilateral mediastinal and left supraclavicular lymph nodes are present. The patient was diagnosed as having adenocarcinoma with high PD‐L1 expression (tumor proportion score = 80%) and epidermal growth factor receptor wild‐type via ultrasound‐guided neck lymph node biopsy. (c) Radiomic feature extraction was performed with segmentation of the volume of interest of the left upper lobe nodule. The Rad‐score is 1.350, which is higher than the cutoff value of −0.715 for positive PD‐L1 expression. CT, computed tomography; PD‐L1, programmed death ligand 1.

Radiomic feature extraction was performed semi‐automatically by two radiologists (one radiology resident and one board‐certificated thoracic radiologist with 2 and 10 years' experience in chest CT imaging, respectively). Digital Imaging and Communications in Medicine (DICOM) files were loaded into a commercialized software (AVIEW Research, Coreline Soft Inc., Seoul, South Korea) and lesion segmentation was performed using a lung window setting (width, 1500 HU; level, ‐600 HU) images (Fig 2). Using the software, the volume of interest (VOI) was delineated around the tumor outline slice by slice on the axial CT images as follows: After importing DICOM files into the software, we used brush tools to manually delineate the VOI slice by slice at the voxel level. Image magnification and three‐dimensional view techniques were used to facilitate precise segmentation. Large vessels and bronchioles were excluded from the VOIs where possible. From a segmented VOI, a total of 58 radiomic features were extracted: 15 histogram features, two gradient features, 13 gray‐level co‐occurrence matrix (GLCM) features, 13 gray‐level run‐length matrix (GLRLM) features, three moment features, 11 shape features, and one fractal features (Table S1).

### PD‐L1 analysis method

Expression of PD‐L1 in histopathologic specimens was determined using the PD‐L1 22C3 pharmDx antibody (Dako North America Inc., Carpinteria, CA, USA) or Ventana PD‐L1 SP263 antibody (Ventana Medical Systems, Tucson, AZ, USA) as a companion diagnosis. Positive tumor cells were defined as complete circumferential or partial cell membrane staining. Cytoplasmic staining and tumor‐associated immune cells (such as macrophages) were excluded from the scoring. Finally, TPS was calculated as a percentage of PD‐L1‐positive tumor cells relative to the total tumor cells. We defined “PD‐L1 expression positive” as 50% or more viable tumor cells exhibiting membrane staining with any intensity (TPS ≥50%).^24^, ^25^ The 74 enrolled patients were divided into two groups by PD‐L1 expression: a “PD‐L1 positive” group and a “PD‐L1 negative” group.

### Statistical analysis

Statistical analysis was performed with SPSS software, version 20.0 (SPSS, Chicago, IL, USA), MedCalc for Windows, version 18.6.0.0 (MedCalc Software, Mariakerke, Belgium), and R (version 3.6.0.; R Foundation for Statistical Computing, Vienna, Austria). Categorical variables are shown as numbers with percentages. Continuous variables are presented as the mean ± standard deviation. Demographics, CT visual analysis results, and CT radiomic features were compared between PD‐L1 positive and PD‐L1 negative groups by chi‐square test for categorical variables, and independent *t*‐test for continuous variables. Interobserver agreements were analyzed using the weighted kappa statistic for qualitative CT features from visual analysis and the intraclass correlation coefficient (ICC) for the lesion diameter and CT radiomic features. Weighted kappa values were interpreted as follows: poor, <0.2; fair, 0.2–0.4; moderate, 0.4–0.6; good, 0.6–0.8; and excellent, >0.8. ICCs were interpreted as follows: poor, <0.5; moderate, 0.5–0.75; good, 0.75–0.9; excellent, >0.9. ICC values lower than zero were considered zero for the analysis.

To diminish the high dimension of the radiomic features to the number of events, we performed three sequential steps for radiomic feature selection. At first, we evaluated the interobserver agreement of radiomic features and selected features showing ICC > 0.75. For the next step, we chose radiomic features which showed statistical significance between the PD‐L1 positive and PD‐L1 negative groups. Finally, the least absolute shrinkage and selection operator (LASSO) logistic regression model was used to choose the most useful predictive features for PD‐L1 positivity: three‐fold cross validation was performed 100 times to avoid the overfitting. Features showing nonzero coefficient were selected when the mean of the calculated area under the receiver operating characteristic (ROC) curve (AUC, predictive accuracy) of LASSO regression model reached maximum among 100 times three‐fold cross validations. A Rad‐score (radiomic score) was calculated for each case via a linear combination of selected features that were weighted by their respective coefficient on the LASSO logistic regression model.^26^

Continuous variables such as age and Rad‐score were dichotomized, and the optimal cutoff value to predict PD‐L1 positivity was calculated from the ROC curves using Youden index. Univariate and multivariate logistic regression analyses were performed to assess the association between clinical variables/CT visual analysis results/Rad‐score and PD‐L1 positivity. We constructed two models for multivariate logistic regression analysis (one based on the clinical variables, and the other based on a combination of clinical variables and imaging features) and compared c‐statistics of each model to identify the model with the higher predictability. For internal validation of the result within the study population, we performed bootstrap validation with 1000 resampling and optimism corrected AUC (c‐index) with 95% confidence interval (CI) was analyzed.^27^ A *P*‐value less than 0.05 less was considered statistically significant.

## Results

### Clinical characteristics of patients

Among 153 patients, 53 patients were classified as PD‐L1 positive and 100 patients were classified as PD‐L1 negative (Table 1). There was no significant difference in clinical characteristics including age, sex, smoking history, TNM stage, and *EGFR* mutation status between the two PD‐L1 expression groups (*P* > 0.05 for all).

**Table 1:** Comparison of demographic features according to PD‐L1 expression

	PD‐L1 expression		
	Positive (*n* = 53)	Negative (*n* = 100)	*P*‐value
Age (mean ± SD)	64.1 ± 11.2	64.8 ± 10.6	0.6916
Sex			0.9170
Male	34 (64.2)	65 (65.0)	
Female	19 (35.8)	35 (35.0)	
Smoking			0.9630
Current smoker	9 (17.0)	18 (18.0)	
Past smoker	20 (37.7)	39 (39.0)	
Never smoker	24 (45.3)	43 (43.0)	
TNM stage			0.3830
3B	5 (9.4)	6 (6.0)	
3C	3 (5.7)	1 (1.0)	
4A	13 (24.5)	27 (27.0)	
4B	32 (60.4)	65 (65.0)	
4C	0 (0)	1 (1.0)	
*EGFR* mutation			0.8061
Yes	18 (34.0)	32 (32.0)	
No	35 (66.0)	68 (68.0)	
Site of histopathologic diagnosis			0.6232
Lung	22 (41.5)	55 (55.0)	
Lymph node	23 (43.4)	34 (34.0)	
Liver	2 (3.8)	3 (3.0)	
Brain	3 (5.7)	4 (4.0)	
Bone	3 (8.7)	3 (3.0)	
Miscellaneous	0 (0)	1 (1.0)	
Method of histopathologic diagnosis (for lung lesion)			0.3089
CT‐guide lung biopsy	6 (27.3)	24 (43.6)	
Transbronchial lung biopsy	16 (72.7)	30 (54.5)	
Video‐assisted thoracoscopic surgery	0 (0.0)	1 (1.8)	

Unless otherwise indicated, data in parentheses are percentages. *EGFR*, epidermal growth factor receptor; PD‐L1, programmed death ligand 1; SD, standard deviation.

### Association between PD‐L1 expression and CT visual analysis

Among imaging findings which were analyzed by visual analysis, none showed a significant difference between the two PD‐L1 expression groups (*P* > 0.05, Table 2).

**Table 2:** Comparison of computed tomography (CT) visual analysis results according to PD‐L1 expression

	PD‐L1 expression		
	Positive (*n* = 53)	Negative (*n* = 100)	*P‐*value
Maximal diameter of tumor (mm) (mean ± SD)	48.1 ± 19.7	44.5 ± 23.2	0.710
Minimal diameter of tumor (mm) (mean ± SD)	31.7 ± 13.6	27.6 ± 14.4	0.937
CT pattern			0.933
Solid predominant part‐solid nodule	3 (5.7)	6 (6.0)	
Pure solid nodule	33 (94.3)	94 (94.0)	
Distribution			0.430
Central	22 (41.5)	35 (35.0)	
Peripheral	31 (58.5)	65 (65.0)	
Lobe location			0.535
Right upper lobe	16 (30.2)	23 (23.0)	
Right middle lobe	4 (7.5)	4 (4.0)	
Right lower lobe	12 (22.6)	25 (25.0)	
Left upper lobe	9 (17.0)	27 (27.0)	
Left lower lobe	12 (22.6)	21 (21.0)	
Contour			0.678
Irregular	3 (5.7)	9 (9.0)	
Round or oval	50 (94.3)	91 (91.0)	
Lobulation	23 (43.4)	41 (41.0)	0.909
Concavity	47 (88.7)	93 (93.0)	0.544
Spiculation	34 (64.2)	75 (75.0)	0.221
Calcification	4 (7.5)	10 (10.0)	0.837
Air bronchogram	20 (37.7)	35 (35.0)	0.874
Bubble‐like lucency	2 (3.8)	5 (5.0)	>0.999
Fissure attachment	25 (47.2)	36 (36.0)	0.242
Pleural attachment	44 (83.0)	77 (77.0)	0.508
Thickened adjacent bronchovascular bundle	23 (44.2)	39 (39.0)	0.654
Pleural retraction	31 (58.5)	62 (62.0)	0.803
Peripheral emphysema	8 (15.1)	13 (13.0)	0.911
Cavitation	4 (7.5)	7 (7.0)	>0.999
Necrosis	23 (43.4)	31 (31.0)	0.177
Pleural effusion	23 (43.4)	33 (33.0)	0.274
N stage			0.228
N0	2 (3.8)	10 (10.0)	
N1	3 (5.7)	11 (11.0)	
N2	14 (26.4)	17 (17.0)	
N3	34 (64.2)	62 (62.0)	
Lesion type			0.489
Mass	36 (67.9)	69 (69.0)	
Nodule	15 (28.3)	28 (28.0)	
Multicentric	0 (0.0)	2 (2.0)	
Consolidation with GGO	2 (3.8)	1 (1.0)	
Lung metastasis			0.313
No	36 (67.9)	53 (53.0)	
Miliary (< 5 mm)	3 (5.7)	4 (4.0)	
Scattered (≥ 5 mm)	6 (11.3)	24 (24.0)	
Lymphangitic	5 (9.4)	13 (13.0)	
Hematolymphangitic	3 (5.7)	6 (6.0)	
Pleural nodularity	25 (47.2)	50 (50.0)	0.87
Significant pericardial effusion	9 (17.0)	9 (9.0)	0.232
Intrathoracic bone metastasis	14 (26.4)	37 (37.0)	0.254

Unless otherwise indicated, data in parentheses are percentages. CT, computed tomography; GGO, ground‐glass opacity; SD, standard deviation.

### Interobserver agreement for visual analysis and radiomic features

Details of interobserver agreement for visual analysis are presented in Table S2. Interobserver agreement for the measurement of maximal and minimal diameters of tumor was excellent (ICC 0.874 and 0.955, respectively). Most of the 25 CT visual analysis features showed good to excellent interobserver agreement (weighted kappa >0.6). Only bubble‐like lucency showed moderate interobserver agreement (weighted kappa = 0.555).

Most of the 58 radiomic features showed good to excellent interobserver agreement (ICC > 0.75). Texture_Histo_Skewness and Texture_GLRLM_RLNUN (run‐length nonuniformity normalized of GLRLM) showed moderate interobserver agreement (ICC 0.5–0.75). Details of the ICCs for all radiomic features are described in Table S3.

### Selection of CT radiomic features

Among CT radiomic features, Texture_GLCM_ASM (angular second momentum of GLCM) and most of GLRLM features showed significant differences between PD‐L1 positive and PD‐L1 negative groups (*P* < 0.05 for all, Table 3). No other CT radiomic feature was significantly different between the two PD‐L1 expression groups (*P* > 0.05).

**Table 3:** Comparison of computed tomography (CT) radiomic features according to PD‐L1 expression

	PD‐L1 expression		
	Positive (*n* = 53)	Negative (*n* = 100)	*P*‐value
Histogram feature (mean ± SD)			
Texture_Histo_Mean (HU)	0.9 ± 73.0	−1.4 ± 78.2	0.861
Texture_Histo_SD (HU)	114.3 ± 65.6	109.8 ± 60.5	0.671
Texture_Histo_Skewness	−2.5 ± 1.9	−2.6 ± 1.5	0.643
Texture_Histo_ExcessKurtosis	20.4 ± 25.6	17.4 ± 17.9	0.448
Texture_Histo_Energy	0.0070 ± 0.0031	0.0063 ± 0.0022	0.124
Texture_Histo_Entropy	7.9 ± 0.7	7.9 ± 0.6	0.567
Texture_Histo_Min (HU)	−825.4 ± 153.1	−807.4 ± 159.3	0.499
Texture_Histo_Max (HU)	378.1 ± 289.8	376.4 ± 268.8	0.971
Texture_Histo_Voxel count	61 292.2 ± 86 630.8	78 247.3 ± 180 123.1	0.433
Percentile (mean ± SD)			
Texture_Percentile_10 (HU)	−136.1 ± 195.6	−133.1 ± 185.1	0.926
Texture_Percentile_25 (HU)	−26.8 ± 107.9	−34.5 ± 117.7	0.695
Texture_Percentile_50 (HU)	32.8 ± 55.6	28.6 ± 67.0	0.695
Texture_Percentile_75 (HU)	65.5 ± 35.3	63.1 ± 39.1	0.71
Texture_Percentile_90 (HU)	90.1 ± 38.8	88.2 ± 34.4	0.766
Texture_Percentile_95 (HU)	105.8 ± 46.5	103.5 ± 36.6	0.763
Gradient feature (mean ± SD)			
Texture_Grad_Mean	119.3 ± 70.4	121.6 ± 70.1	0.848
Texture_Grad_SD	124.9 ± 39.4	122.5 ± 38.0	0.714
GLCM feature (mean ± SD)			
Texture_GLCM_ASM	0.0737 ± 0.0478	0.0582 ± 0.0331	0.038
Texture_GLCM_IDM	0.5 ± 0.1	0.5 ± 0.1	0.243
Texture_GLCM_Homogeneity	0.6 ± 0.1	0.6 ± 0.1	0.242
Texture_GLCM_Contrast	9.2 ± 10.6	8.6 ± 9.0	0.744
Texture_GLCM_Correlation	0.6 ± 0.2	0.6 ± 0.1	0.985
Texture_GLCM_Autocor	1094.4 ± 120.4	1090.8 ± 128.5	0.868
Texture_GLCM_Entropy	5.3 ± 1.4	5.4 ± 1.1	0.497
Texture_GLCM_CP	48 607.0 ± 148 803.9	32 033.0 ± 87 988.2	0.459
Texture_GLCM_CS	−1028.0 ± 2715.2	−750.4 ± 1673.0	0.499
Texture_GLCM_CT	51.5 ± 87.7	44.5 ± 67.1	0.612
Texture_GLCM_SumEntropy	3.7 ± 0.8	3.7 ± 0.7	0.635
Texture_GLCM_DiffAverage,	1.7 ± 1.0	1.7 ± 0.9	0.925
Texture_GLCM_DiffEntropy	2.3 ± 0.6	2.3 ± 0.5	0.721
GLRLM feature (mean ± SD)			
Texture_GLRLM_SRE	0.0494 ± 0.0285	0.0347 ± 0.0229	0.001
Texture_GLRLM_LRE	0.5 ± 0.6	0.2 ± 0.3	0.002
Texture_GLRLM_LGRE	0.0003 ± 0.0012	0.0001 ± 0.0008	0.471
Texture_GLRLM_HGRE	89.1 ± 59.2	57.6 ± 42.3	0.001
Texture_GLRLM_SRLGE	0.0002 ± 0.0007	0.0001 ± 0.0005	0.485
Texture_GLRLM_SRHGE	56.8 ± 33.8	39.7 ± 26.8	0.002
Texture_GLRLM_LRLGE	0.0020 ± 0.0106	0.0008 ± 0.0058	0.454
Texture_GLRLM_LRHGE	604.0 ± 722.4	266.7 ± 319.9	0.002
Texture_GLRLM_GNUN	0.0034 ± 0.0044	0.0013 ± 0.0020	0.002
Texture_GLRLM_RLNUN	0.0031 ± 0.0030	0.0016 ± 0.0019	0.001
Texture_GLRLM_RP	0.0771 ± 0.0503	0.0501 ± 0.0360	0.001
Texture_GLRLM_RV	0.4 ± 0.5	0.2 ± 0.2	0.002
Texture_GLRLM_RE	0.5 ± 0.3	0.4 ± 0.2	0.001
Moment feature (mean ± SD)			
Texture_Moment_J1	29.0 ± 46.4	32.9 ± 67.6	0.669
Texture_Moment_J2	0.0007 ± 0.0022	0.0015 ± 0.0054	0.221
Texture_Moment_J3	<0.0001 ± <0.0001	<0.0001 ± <0.0001	0.203
Shape feature (mean ± SD)			
Shape_Volume (mm^3^)	43 718.0 ± 53 318.6	51 640.9 ± 114 615.3	0.561
Shape_SurfaceArea (mm^2^)	11 496.3 ± 10 465.8	12 840.4 ± 15 782.6	0.530
Shape_Sphericity	0.5 ± 0.1	0.5 ± 0.1	0.417
Shape_Compactness	1.0 ± 0.0	0.9 ± 0.0	0.426
Shape_Roundness	0.7 ± 0.1	0.7 ± 0.1	0.089
Shape_Circularity	0.4 ± 0.1	0.3 ± 0.1	0.442
Shape_Longest1stAxis (mm)	54.2 ± 22.4	57.8 ± 30.9	0.408
Shape_Longest2ndAxis (mm)	44.0 ± 19.1	45.3 ± 23.1	0.726
Shape_PCA1stMajorSD (mm)	11.3 ± 4.7	11.7 ± 6.3	0.626
Shape_PCA2ndMajorSD (mm)	8.6 ± 3.6	8.7 ± 4.3	0.823
Shape_PCA3rdMajorSD (mm)	7.1 ± 3.0	7.0 ± 3.6	0.779
Fractal feature (mean ± SD)			
Fractal dimension	2.4 ± 0.2	2.4 ± 0.2	0.626

Unless otherwise indicated, data in parentheses are percentages. ASM, angular second moment; Autocor, autocorrelation; CP, cluster prominence; CS, cluster shade; CT, cluster tendency; GLCM, gray‐level co‐occurrence matrix; GLRLM, gray‐level run‐length matrix; GNUN, gray‐level nonuniformity normalized; Grad, gradient; HGRE, high gray‐level run emphasis; Histo, histogram; HU, Hounsfield Unit; IDM, inverse different moment; LGRE, low gray‐level run emphasis; LRE, long run emphasis; LRHGE, long run high gray‐level emphasis; LRLGE, long run low gray‐level emphasis; Max, Maximum; Min, minimum; PCA, principal component analysis; PD‐L1 = programmed death ligand 1; RE, run entropy; RLNUN, run‐length nonuniformity normalized; RP, run percentage; RV, run variance; SD, standard deviation; SRE, short run emphasis; SRHGE, short run high gray‐level emphasis; SRLGE, short run low gray‐level emphasis.

After feature selection processes, selected radiomics feature sets were as follows: Texture_GLCM_ASM, Texture_GLRLM_RV (run variance of GLRLM), Texture_GLRLM_RE (run entropy of GLRLM), Texture_GLRLM_SRHGE (short‐run high gray‐level emphasis of GLRLM). The radiomics signature was computed into a Rad‐score by using the following formula:

Rad‐score = −(1.594 23) + Texture_GLCM_ASM x − 8.495 68 + Texture_GLRLM_RV x 3.585 97 + Texture_GLRLM_RE x (−5.01416) + Texture_GLRLM_SRHGE x 0.05253.

The Rad‐score was higher in the PD‐L1 positive group than in the PD‐L1 negative group with a statistical significance (−0.378 ± 1.537 vs. −1.171 ± 0.822, *P* = 0.0008). The AUC of Rad‐score to predict PD‐L1 positivity was 0.661 (95% CI 0.580–0.735) and the optimum cutoff value calculated from the ROC curves was −0.715 (sensitivity 52.8%, specificity 76.0%). In patients with *EGFR* wild‐type tumor, the Rad‐score was higher in the PD‐L1 positive group than in the PD‐L1 negative group with a statistical significance (−0.419 ± 1.578 vs. −1.135 ± 0.861, *P* = 0.0162).

### Prediction model for PD‐L1 positivity

In univariate logistic regression analysis, a Rad‐score > −0.715 showed significant association with PD‐L1 status (odds ratio [OR] 3.3600; 95% CI 1.6617–6.7940; *P* = 0.0007; c‐statistic 0.639 [95% CI 0.558–0.715]; Table 4). None of the clinical variables or qualitative imaging features showed a significant association with PD‐L1 status (*P* > 0.05).

**Table 4:** Univariate logistic regression analysis for prediction of PD‐L1 positivity

Clinical variables	OR (95% CI)	*P*‐value
Age (≤59 years)	0.7299 (0.3480–1.5266	0.4017
Female sex	1.0378 (0.5176–2.0810)	0.9167
Current or ex‐smoker	0.9512 (0.4841–1.8690)	0.8845
Presence of *EGFR* mutation	1.0929 (0.5390–2.2160)	0.8055
Rad‐score		
Rad‐score > −0.715	3.3600 (1.6617–6.7940)	0.0007

CI, confidence interval; *EGFR*, epidermal growth factor receptor; OR, odds ratio; PD‐L1, programmed death ligand 1.

We established two prediction models for predicting PD‐L1 positivity: model 1 uses clinical variables and model 2 uses clinical variables and CT radiomic features. The predictive performance was higher with model 2 (c‐statistic = 0.667; 95% CI = 0.575–0.760) than model 1 (c‐statistic = 0.550; 95% CI = 0.454–0.646), with a statistical significance (*P* = 0.0299, Table 5). The c‐statistics in the development set were similar to the values with bootstrap estimates in the internal validation, with significant difference between two models (difference of c‐statistics between two models, 0.117, 95% CI = 0.012–0.225).

**Table 5:** Multivariate logistic regression models for prediction of PD‐L1 positivity

	OR	95% CI	*P*‐value		OR	95% CI	*P*‐value
Model 1 (clinical variables)				Model 2 (clinical variables + CT radiomic features)			
Age ≤ 59 years	0.7281	0.3362–1.5232	0.408	0.6106		0.2691–1.3259	0.2227
Female sex	1.0820	0.3793–3.1603	0.883	0.9354		0.3121–2.8772	0.9053
Current or ex‐smoker	1.0635	0.3823–3.1009	0.907	0.9115		0.3129–2.7532	0.8658
Presence of *EGFR* mutation	1.1710	0.5272–2.5799	0.695	1.1098		0.4821–2.5304	0.8042
Rad‐score > −0.715	N/A	N/A	N/A	3.4706		1.6919–7.2840	0.0008
C‐statistic (95% CI)	0.550 (0.454–0.646)			0.667 (0.575–0.760)		*P*‐value for comparison of c‐statistics between two models	0.0299
Bootstrapped c‐statistic (95% CI)	0.550 (0.461–0.6488)			0.667 (0.577–0.764)			

CI, confidence interval; *EGFR*, epidermal growth factor receptor; OR, odds ratio; PD‐L1, programmed death ligand 1.

## Discussion

Our study demonstrates that quantitative radiomic features can help predict PD‐L1 expression in advanced lung adenocarcinoma, whereas none of the qualitative imaging findings is associated with PD‐L1 positivity. Furthermore, a prediction model constructed with Rad‐score in combination with clinical variables shows a higher c‐statistic than a model constructed with clinical variables only.

Since PD‐L1 has been expected to predict the response of immune checkpoint inhibitors in lung cancer patients,^8^, ^9^, ^10^ there were few previous studies that attempted to predict PD‐L1 expression noninvasively in surgically resected lung adenocarcinomas using imaging modalities.^12^, ^13^, ^28^ Previous studies reported that qualitative CT features such as lobular/irregular shape, pleural indentation, presence of convergence/cavitation, absence of surrounding GGO/air‐bronchogram, and quantitative CT imaging features such as mean CT attenuation of tumor, higher consolidation to tumor mass ratio (C/T ratio), and higher maximum standardized uptake value on positron emission tomography were significantly associated with PD‐L1 positivity.^12^, ^13^, ^28^

According to previous studies regarding imaging features of PD‐L1‐positive NSCLCs, a large solid portion with a small GGO on CT scan was a common feature associated with PD‐L1 expression, which can be explained by a correlation with pathological invasiveness, histologic subtype, or proportion of *EGFR* mutation.^12^, ^13^, ^28^ In surgically‐resected lung adenocarcinomas, tumors with PD‐L1 expression tended to be more invasive histologic subtypes with a worse prognosis (e.g., solid predominant) than tumors without PD‐L1 expression.^12^, ^13^, ^28^, ^29^ Because GGO in subsolid nodules is thought to correlate with the lepidic component of lung adenocarcinomas, lung adenocarcinomas with preinvasive or lepidic predominant subtypes mostly present as pure ground‐glass nodules or part‐solid nodules on CT, whereas lung adenocarcinomas with micropapillary or solid predominant subtypes present as pure solid nodules.^30^, ^31^, ^32^, ^33^, ^34^ Meanwhile, NSCLCs with *EGFR* mutations tended to have higher GGO proportions on CT,^31^, ^34^, ^35^, ^36^, ^37^, ^38^ which might be explained by the fact that they have a high prevalence of lepidic‐predominant histologic types.^31^, ^39^, ^40^, ^41^, ^42^, ^43^ The presence of an *EGFR* mutation was thought to be inversely correlated with PD‐L1 expression in NSCLCs,^44^ although there have been controversies, and the relationship was not statistically significant in our study. Therefore, a large solid portion with a small GGO on CT in a PD‐L1 positive adenocarcinoma might demonstrate the relationship between CT findings with histologic subtype, and also with *EGFR* mutation.

Other qualitative CT features including lobular/irregular shape, presence of convergence/cavitation, and pleural indentation have been suggested as predictive imaging features of PD‐L1 positivity and were also supposed to be associated with the pathological invasiveness of the tumor. However, in our study, none of the qualitative imaging features on visual analysis was related to PD‐L1 positivity. This result may be due to differences in the clinical characteristics of our study population compared to those in previous studies. Previous studies also included patients with surgically resected lung adenocarcinomas, the majority of which were early stage, resectable cases.^12^, ^13^, ^28^ On the other hand, our study included patients with unresectable adenocarcinomas, who could be better candidates for immunotherapy than patients with early stage tumors.^45^ Therefore, the results of our study may have more clinical value than those of previous studies.

Although interest in quantitative imaging biomarker is increasing, the application of radiomics in thoracic oncology has been limited to prediction of *EGFR* mutation or survival after treatment.^14^, ^15^, ^16^, ^17^, ^18^, ^19^ Our study suggests that adding radiomic features to clinical variables could increase predictability for PD‐L1 expression in advanced lung adenocarcinomas, and to our knowledge, this was the first attempt to investigate the value of radiomic features for prediction of PD‐L1 expression.

In our study, four radiomic features (Texture_GLCM_ASM, Texture_GLRLM_RV, Texture_GLRLM_RE, Texture_GLRLM_SRHGE) were selected. Texture_GLCM_ASM is a measure of homogenous patterns in the image, and GLRLM quantifies gray level runs, which are defined as the length of consecutive voxels that have the same gray level value. Since the Rad‐scores in our study demonstrated a tendency for larger Texture_GLCM_ASM, Texture_GLRLM_RV and Texture_GLRLM_SRHGE with smaller Texture_GLRLM_RE being correlated with PD‐L1 expression, the lesion with homogenous and high CT attenuating large voxel values could be more likely to be PD‐L1‐positive. In other words, a homogenous tumor presenting as a pure solid nodule with no or small GGO, inner necrosis, cavitation, or calcification may have PD‐L1 positivity in advanced lung adenocarcinoma, which was similar to the results of previous studies of early stage lung adenocarcinomas, even though the trend was not clearly seen on visual analysis in our study.

This study had several limitations. First, it was conducted retrospectively from a single tertiary referral center, and patients were identified only from those having PD‐L1 testing results, which can lead to a selection bias. Second, the proposed prediction model did not undergo external validation in other cohorts, therefore, our findings might be difficult to generalize. Third, the PD‐L1 test lacks universal reference standards, and among several testing methods for confirming PD‐L1 positivity,^46^ PD‐L1 immunohistochemistry was conducted with only two antibodies and one cutoff value. Finally, the treatment response after immunotherapy was not assessed. Further studies are needed to evaluate the predictive value of CT radiomic features for treatment response after anti‐PD‐L1 therapy.

In conclusion, quantitative CT radiomic features can predict PD‐L1 expression in advanced stage lung adenocarcinoma. Furthermore, a prediction model composed of clinical variables and CT radiomic features may facilitate noninvasive assessment of PD‐L1 expression.

## Disclosure

The authors declare that there are no conflicts of interest.

## Supporting information


**Table S1.** Extracted radiomic features by feature category.
**Table S2.** Interobserver variability for CT visual analysis.
**Table S3.** Interobserver variability for CT radiomic features.

## References

[1] Ferlay J , Soerjomataram I , Dikshit R *et al* Cancer incidence and mortality worldwide: Sources, methods and major patterns in GLOBOCAN 2012. Int J Cancer 2015; 136: E359–86. 25220842 10.1002/ijc.29210

[2] Torre LA , Bray F , Siegel RL , Ferlay J , Lortet‐Tieulent J , Jemal A . Global cancer statistics, 2012. CA Cancer J Clin 2015; 65: 87–108. 25651787 10.3322/caac.21262

[3] Hirsch FR , Scagliotti GV , Mulshine JL *et al* Lung cancer: Current therapies and new targeted treatments. Lancet 2017; 389: 299–311. 27574741 10.1016/S0140-6736(16)30958-8

[4] Ai X , Guo X , Wang J *et al* Targeted therapies for advanced non‐small cell lung cancer. Oncotarget 2018; 9: 37589–607. 30680072 10.18632/oncotarget.26428 PMC6331020

[5] Borghaei H , Paz‐Ares L , Horn L *et al* Nivolumab versus Docetaxel in advanced nonsquamous non‐small‐cell lung cancer. N Engl J Med 2015; 373: 1627–39. 26412456 10.1056/NEJMoa1507643 PMC5705936

[6] Brahmer J , Reckamp KL , Baas P *et al* Nivolumab versus Docetaxel in advanced squamous‐cell non‐small‐cell lung cancer. N Engl J Med 2015; 373: 123–35. 26028407 10.1056/NEJMoa1504627 PMC4681400

[7] Reck M , Rodriguez‐Abreu D , Robinson AG *et al* Pembrolizumab versus chemotherapy for PD‐L1‐positive non‐small‐cell lung cancer. N Engl J Med 2016; 375: 1823–33. 27718847 10.1056/NEJMoa1606774

[8] Takada K , Toyokawa G , Okamoto T *et al* A comprehensive analysis of programmed cell death Ligand‐1 expression with the clone SP142 antibody in non‐small‐cell lung cancer patients. Clin Lung Cancer 2017; 18: 572–82. 28318951 10.1016/j.cllc.2017.02.004

[9] Sacher AG , Gandhi L . Biomarkers for the clinical use of PD‐1/PD‐L1 inhibitors in non‐small‐cell lung cancer: A review. JAMA Oncol 2016; 2: 1217–22. 27310809 10.1001/jamaoncol.2016.0639

[10] Takada K , Okamoto T , Shoji F *et al* Clinical significance of PD‐L1 protein expression in surgically resected primary lung adenocarcinoma. J Thorac Oncol 2016; 11: 1879–90. 27346415 10.1016/j.jtho.2016.06.006

[11] Tsao MS , Kerr KM , Dacic S , Yatabe Y , Hirsch F . IASLC Atlas of PD‐L1 Immunohistochemistry Testing in Lung Cancer. International Association for the Study of Lung Cancer, Aurora, CO 2017.

[12] Toyokawa G , Takada K , Okamoto T *et al* Relevance between programmed death ligand 1 and radiologic invasiveness in pathologic stage I lung adenocarcinoma. Ann Thorac Surg 2017; 103: 1750–7. 28347537 10.1016/j.athoracsur.2016.12.025

[13] Wu T , Zhou F , Soodeen‐Lalloo AK *et al* The association between imaging features of TSCT and the expression of PD‐L1 in patients with surgical resection of lung adenocarcinoma. Clin Lung Cancer 2019; 20: e195–207. 30514666 10.1016/j.cllc.2018.10.012

[14] Jia TY , Xiong JF , Li XY *et al* Identifying EGFR mutations in lung adenocarcinoma by noninvasive imaging using radiomics features and random forest modeling. Eur Radiol 2019; 29: 4742–50. 10.1007/s00330-019-06024-y. 30778717

[15] Digumarthy SR , Padole AM , Gullo RL , Sequist LV , Kalra MK . Can CT radiomic analysis in NSCLC predict histology and EGFR mutation status? Medicine 2019; 98: e13963. 30608433 10.1097/MD.0000000000013963 PMC6344142

[16] Mei D , Luo Y , Wang Y , Gong J . CT texture analysis of lung adenocarcinoma: Can radiomic features be surrogate biomarkers for EGFR mutation statuses. Cancer Imaging 2018; 18: 52. 30547844 10.1186/s40644-018-0184-2 PMC6295009

[17] Ozkan E , West A , Dedelow JA *et al* CT gray‐level texture analysis as a quantitative imaging biomarker of epidermal growth factor receptor mutation status in adenocarcinoma of the lung. AJR Am J Roentgenol 2015; 205: 1016–25. 26496549 10.2214/AJR.14.14147

[18] Liu Y , Kim J , Balagurunathan Y *et al* Radiomic features are associated with EGFR mutation status in lung adenocarcinomas. Clin Lung Cancer 2016; 17: 441–448.e6. 27017476 10.1016/j.cllc.2016.02.001 PMC5548419

[19] Rizzo S , Petrella F , Buscarino V *et al* CT radiogenomic characterization of EGFR, K‐RAS, and ALK mutations in non‐small cell lung cancer. Eur Radiol 2016; 26: 32–42. 25956936 10.1007/s00330-015-3814-0

[20] Goldstraw P , Chansky K , Crowley J *et al* The IASLC lung cancer staging project: Proposals for revision of the TNM stage groupings in the forthcoming (eighth) edition of the TNM classification for lung cancer. J Thorac Oncol 2016; 11: 39–51. 26762738 10.1016/j.jtho.2015.09.009

[21] Chang S , Hur J , Hong YJ *et al* Adverse prognostic CT findings for patients with advanced lung adenocarcinoma receiving first‐line epidermal growth factor receptor‐tyrosine kinase inhibitor therapy. AJR Am J Roentgenol 2018; 210: 43–51. 29091002 10.2214/AJR.17.18167

[22] Liu Y , Kim J , Qu F *et al* CT features associated with epidermal growth factor receptor mutation status in patients with lung adenocarcinoma. Radiology 2016; 280: 271–80. 26937803 10.1148/radiol.2016151455 PMC4934516

[23] Choi CM , Kim MY , Hwang HJ , Lee JB , Kim WS . Advanced adenocarcinoma of the lung: Comparison of CT characteristics of patients with anaplastic lymphoma kinase gene rearrangement and those with epidermal growth factor receptor mutation. Radiology 2015; 275: 272–9. 25575117 10.1148/radiol.14140848

[24] Garon EB , Rizvi NA , Hui R *et al* Pembrolizumab for the treatment of non‐small‐cell lung cancer. N Engl J Med 2015; 372: 2018–28. 25891174 10.1056/NEJMoa1501824

[25] McLaughlin J , Han G , Schalper KA *et al* Quantitative assessment of the heterogeneity of PD‐L1 expression in non‐small‐cell lung cancer. JAMA Oncol 2016; 2: 46–54. 26562159 10.1001/jamaoncol.2015.3638 PMC4941982

[26] Huang Y , Liu Z , He L *et al* Radiomics signature: A potential biomarker for the prediction of disease‐free survival in early‐stage (I or II) non‐small cell lung cancer. Radiology 2016; 281: 947–57. 27347764 10.1148/radiol.2016152234

[27] Han K , Song K , Choi BW . How to develop, validate, and compare clinical prediction models involving radiological parameters: Study design and statistical methods. Korean J Radiol 2016; 17: 339–50. 27134523 10.3348/kjr.2016.17.3.339 PMC4842854

[28] Toyokawa G , Takada K , Okamoto T *et al* Computed tomography features of lung adenocarcinomas with programmed death ligand 1 expression. Clin Lung Cancer 2017; 18: e375–83. 28385373 10.1016/j.cllc.2017.03.008

[29] Shimoji M , Shimizu S , Sato K *et al* Clinical and pathologic features of lung cancer expressing programmed cell death ligand 1 (PD‐L1). Lung Cancer 2016; 98: 69–75. 27393509 10.1016/j.lungcan.2016.04.021

[30] Cha MJ , Lee HY , Lee KS *et al* Micropapillary and solid subtypes of invasive lung adenocarcinoma: Clinical predictors of histopathology and outcome. J Thorac Cardiovasc Surg 2014; 147: 921–928.E922. 24199757 10.1016/j.jtcvs.2013.09.045

[31] Lee HJ , Kim YT , Kang CH *et al* Epidermal growth factor receptor mutation in lung adenocarcinomas: Relationship with CT characteristics and histologic subtypes. Radiology 2013; 268: 254–64. 23468578 10.1148/radiol.13112553

[32] Kudo Y , Matsubayashi J , Saji H *et al* Association between high‐resolution computed tomography findings and the IASLC/ATS/ERS classification of small lung adenocarcinomas in Japanese patients. Lung Cancer 2015; 90: 47–54. 26259875 10.1016/j.lungcan.2015.07.007

[33] Ko JP , Suh J , Ibidapo O *et al* Lung adenocarcinoma: Correlation of quantitative CT findings with pathologic findings. Radiology 2016; 280: 931–9. 27097236 10.1148/radiol.2016142975

[34] Yang Y , Yang Y , Zhou X *et al* EGFR L858R mutation is associated with lung adenocarcinoma patients with dominant ground‐glass opacity. Lung Cancer 2015; 87: 272–7. 25582278 10.1016/j.lungcan.2014.12.016

[35] Yano M , Sasaki H , Kobayashi Y *et al* Epidermal growth factor receptor gene mutation and computed tomographic findings in peripheral pulmonary adenocarcinoma. J Thorac Oncol 2006; 1: 413–6. 17409892

[36] Usuda K , Sagawa M , Motono N *et al* Relationships between EGFR mutation status of lung cancer and preoperative factors ‐ Are they predictive? Asian Pac J Cancer Prev 2014; 15: 657–62. 24568474 10.7314/apjcp.2014.15.2.657

[37] Sabri A , Batool M , Xu Z , Bethune D , Abdolell M , Manos D . Predicting EGFR mutation status in lung cancer: Proposal for a scoring model using imaging and demographic characteristics. Eur Radiol 2016; 26: 4141–7. 27027313 10.1007/s00330-016-4252-3

[38] Zou J , Lv T , Zhu S *et al* Computed tomography and clinical features associated with epidermal growth factor receptor mutation status in stage I/II lung adenocarcinoma. Thorac Cancer 2017; 8: 260–70. 28383802 10.1111/1759-7714.12436 PMC5415462

[39] Travis WD , Brambilla E , Noguchi M *et al* International association for the study of lung cancer/american thoracic society/european respiratory society international multidisciplinary classification of lung adenocarcinoma. J Thorac Oncol 2011; 6: 244–85. 21252716 10.1097/JTO.0b013e318206a221 PMC4513953

[40] Yanagawa N , Shiono S , Abiko M , Ogata SY , Sato T , Tamura G . The correlation of the International Association for the Study of Lung Cancer (IASLC)/American Thoracic Society (ATS)/European Respiratory Society (ERS) classification with prognosis and EGFR mutation in lung adenocarcinoma. Ann Thorac Surg 2014; 98: 453–8. 24961844 10.1016/j.athoracsur.2014.04.108

[41] Villa C , Cagle PT , Johnson M *et al* Correlation of EGFR mutation status with predominant histologic subtype of adenocarcinoma according to the new lung adenocarcinoma classification of the International Association for the Study of Lung Cancer/American Thoracic Society/European Respiratory Society. Arch Pathol Lab Med 2014; 138: 1353–7. 24571650 10.5858/arpa.2013-0376-OA

[42] Mansuet‐Lupo A , Bobbio A , Blons H *et al* The new histologic classification of lung primary adenocarcinoma subtypes is a reliable prognostic marker and identifies tumors with different mutation status: The experience of a French cohort. Chest 2014; 146: 633–43. 24676429 10.1378/chest.13-2499

[43] Nakamura H , Saji H , Shinmyo T *et al* Association of IASLC/ATS/ERS histologic subtypes of lung adenocarcinoma with epidermal growth factor receptor mutations in 320 resected cases. Clin Lung Cancer 2015; 16: 209–15. 25467929 10.1016/j.cllc.2014.10.004

[44] Zhang M , Li G , Wang Y *et al* PD‐L1 expression in lung cancer and its correlation with driver mutations: A meta‐analysis. Sci Rep 2017; 7: 10255. 28860576 10.1038/s41598-017-10925-7 PMC5578960

[45] Sholl L . Molecular diagnostics of lung cancer in the clinic. Transl Lung Cancer Res 2017; 6: 560–9. 29114472 10.21037/tlcr.2017.08.03 PMC5653520

[46] Kim H , Chung JH . PD‐L1 testing in non‐small cell lung cancer: Past, present, and future. J Pathol Transl Med 2019; 53: 199–206. 10.4132/jptm.2019.04.24. 31042863 PMC6639705

