# Supplementary material for: Utility of CT radiomics for prediction of PD‐L1 expression in advanced lung adenocarcinomas
Source: Thorac Cancer. 2020 Feb 11;11(4):993–1004. doi: 10.1111/1759-7714.13352 (PMC7113038; doi:10.1111/1759-7714.13352)
Supplement: Supplementary file 1 — Table S1. Extracted radiomic features by feature category. Table S2. Interobserver variability for CT visual analysis. Table S3. Interobserver variability for CT radiomic features. [file TCA-11-993-s001.docx]

**Supplementary Material**

Table 1. Extracted radiomic features by feature category

| Feature category | Extracted CT radiomic features |
| --- | --- |
| Histogram features (15 features) | Texture_Histo_Mean  Texture_Histo_SD  Texture_Histo_Skewness  Texture_Histo_ExcessKurtosis  Texture_Histo_Energy  Texture_Histo_Entropy  Texture_Histo_Min  Texture_Histo_Max  Texture_Histo_VoxelCount  Texture_Percentile_10  Texture_Percentile_25  Texture_Percentile_50  Texture_Percentile_75  Texture_Percentile_90  Texture_Percentile_95 |
| Gradient Features (2 features) | Texture_Grad_Mean  Texture_Grad_SD |
| GLCM Features (13 features) | Texture_GLCM_ASM  Texture_GLCM_IDM  Texture_GLCM_Homogeneity  Texture_GLCM_Contrast  Texture_GLCM_Correlation  Texture_GLCM_Autocor  Texture_GLCM_Entropy  Texture_GLCM_CP  Texture_GLCM_CS  Texture_GLCM_CT  Texture_GLCM_SumEntropy  Texture_GLCM_DiffAverage  Texture_GLCM_DiffEntropy |
| GLRLM Features (13 features) | Texture_GLRLM_SRE  Texture_GLRLM_LRE  Texture_GLRLM_LGRE  Texture_GLRLM_HGRE  Texture_GLRLM_SRLGE  Texture_GLRLM_SRHGE  Texture_GLRLM_LRLGE  Texture_GLRLM_LRHGE  Texture_GLRLM_GNUN  Texture_GLRLM_RLNUN  Texture_GLRLM_RP  Texture_GLRLM_RV  Texture_GLRLM_RE |
| Moment Features (3-features) | Texture_Moment_J1  Texture_Moment_J2  Texture_Moment_J3 |
| Shape Features (11 features) | Shape_Volume  Shape_SurfaceArea  Shape_Sphericity  Shape_Compactness  Shape_Roundness  Shape_Circularity  Shape_Longest1stAxis  Shape_Longest2ndAxis  Shape_PCA1stMajorSD  Shape_PCA2ndMajorSD  Shape_PCA3rdMajorSD |
| Fractal Features (1 feature) | FractalDimension |

CT, computed tomography; Histo, histogram; Min, minimum; Max, Maximum; Grad, gradient; GLCM, grey-level co-occurrence matrix; ASM, angular second moment; IDM, inverse different moment; Autocor, autocorrelation; CP, cluster prominence; CS, cluster shade; CT, cluster tendency; GLRLM,; grey-level run-length matrix; SRE, short run emphasis; LRE, long run emphasis; LGRE, low gray-level run emphasis; HGRE, high gray-level run emphasis; SRLGE, short run low gray-level emphasis; SRHGE, short run high gray-level emphasis; LRLGE, long run low gray-level emphasis; LRHGE, long run high gray-level emphasis; GNUN, gray-level non-uniformity normalized; RLNUN, run-length non-uniformity normalized; RP, run percentage; RV, run variance; RE, run entropy; PCA, principal component analysis

Table 2. Interobserver variability for CT visual analysis

|  | **Weighted kappa** | **95% confidence interval** |
| --- | --- | --- |
| Maximal diameter of tumor^*^ | 0.874 | 0.798-0.932 |
| Minimal diameter of tumor^*^ | 0.955 | 0.928-0.972 |
| CT pattern | 1.000 | 1.000-1.000 |
| Distribution | 0.734 | 0.581-0.887 |
| Location | 0.992 | 0.976-1.000 |
| Lobulation | 0.839 | 0.715-0.962 |
| Concavity | 0.778 | 0.537-1.000 |
| Spiculation | 0.850 | 0.723-0.976 |
| Calcification | 1.000 | 1.000-1.000 |
| Air bronchogram | 0.804 | 0.665-0.942 |
| Bubble like lucency | 0.555 | 0.112-0.997 |
| Fissure attachment | 0.974 | 0.922-1.000 |
| Pleural attachment | 0.957 | 0.875-1.000 |
| Thickened adjacent bronchovascular bundles | 0.791 | 0.634-0.948 |
| Pleural retraction | 0.760 | 0.613-0.907 |
| Peripheral emphysema | 0.670 | 0.403-0.938 |
| Cavitation | 0.819 | 0.577-1.000 |
| Necrosis | 0.843 | 0.724-0.962 |
| Pleural effusion | 0.921 | 0.833-1.000 |
| Lymphadenopathy | 0.941 | 0.872-1.000 |
| Lesion type | 0.899 | 0.774-1.000 |
| Lung metastasis pattern | 0.754 | 0.608-0.900 |
| Pleural nodularity | 0.733 | 0.583-0.884 |
| Pericardial metastasis | 0.737 | 0.390-1.000 |
| Pericardial effusion | 0.713 | 0.533-0.893 |
| Intrathoracic bone metastasis | 0.910 | 0.810-1.000 |

*Data indicate intraclass correlation coefficient.

CT, computed tomography

Table 3. Interobserver variability for CT radiomic features

|  | **Intraclass correlation coefficient** | **95% confidence interval** |
| --- | --- | --- |
| Histogram feature |  |  |
| Texture_Histo_Mean | 0.962 | 0.935-0.978 |
| Texture_Histo_SD | 0.915 | 0.855-0.951 |
| Texture_Histo_Skewness | 0.735 | 0.582-0.837 |
| Texture_Histo_ExcessKurtosis | 0.868 | 0.781-0.921 |
| Texture_Histo_Energy | 0.984 | 0.972-0.991 |
| Texture_Histo_Entropy | 0.969 | 0.946-0.982 |
| Texture_Histo_Min | 0.767 | 0.630-0.859 |
| Texture_Histo_Max | 0.860 | 0.769-0.917 |
| Texture_Histo_VoxelCount | 0.995 | 0.992-0.997 |
| Texture_Percentile_10 | 0.955 | 0.923-0.973 |
| Texture_Percentile_25 | 0.944 | 0.904-0.967 |
| Texture_Percentile_50 | 0.970 | 0.949-0.983 |
| Texture_Percentile_75 | 0.987 | 0.978-0.993 |
| Texture_Percentile_90 | 0.993 | 0.988-0.996 |
| Texture_Percentile_95 | 0.985 | 0.974-0.991 |
| Gradient feature |  |  |
| Texture_Grad_Mean | 0.973 | 0.951-0.985 |
| Texture_Grad_SD | 0.954 | 0.922-0.973 |
| GLCM Feature |  |  |
| Texture_GLCM_ASM | 0.991 | 0.984-0.995 |
| Texture_GLCM_IDM | 0.986 | 0.976-0.992 |
| Texture_GLCM_Homogeneity | 0.986 | 0.976-0.992 |
| Texture_GLCM_Contrast | 0.976 | 0.968-0.986 |
| Texture_GLCM_Correlation | 0.834 | 0.730-0.901 |
| Texture_GLCM_Autocor | 0.970 | 0.948-0.983 |
| Texture_GLCM_Entropy | 0.976 | 0.959-0.986 |
| Texture_GLCM_CP | 0.867 | 0.780-0.921 |
| Texture_GLCM_CS | 0.857 | 0.765-0.914 |
| Texture_GLCM_CT | 0.918 | 0.862-0.952 |
| Texture_GLCM_SumEntropy | 0.967 | 0.944-0.982 |
| Texture_GLCM_DiffAverage | 0.979 | 0.963-0.988 |
| Texture_GLCM_DiffEntropy | 0.971 | 0.950-0.983 |
| GLRLM Feature |  |  |
| Texture_GLRLM_SRE | 0.761 | 0.619-0.855 |
| Texture_GLRLM_LRE | 0.932 | 0.884-0.960 |
| Texture_GLRLM_LGRE | 0.848 | 0.750-0.910 |
| Texture_GLRLM_HGRE | 0.812 | 0.694-0.887 |
| Texture_GLRLM_SRLGE | 0.866 | 0.778-0.920 |
| Texture_GLRLM_SRHGE | 0.777 | 0.641-0.864 |
| Texture_GLRLM_LRLGE | 0.928 | 0.878-0.958 |
| Texture_GLRLM_LRHGE | 0.934 | 0.887-0.961 |
| Texture_GLRLM_GNUN | 0.755 | 0.610-0.851 |
| Texture_GLRLM_RLNUN | 0.650 | 0.464-0.782 |
| Texture_GLRLM_RP | 0.801 | 0.677-0.880 |
| Texture_GLRLM_RV | 0.944 | 0.903-0.967 |
| Texture_GLRLM_RE | 0.825 | 0.714-0.896 |
| Moment Feature |  |  |
| Texture_Moment_J1 | 0.992 | 0.985-0.995 |
| Texture_Moment_J2 | 0.968 | 0.945-0.982 |
| Texture_Moment_J3 | 0.957 | 0.885-0.985 |
| Shape Feature |  |  |
| Shape_Volume | 0.997 | 0.995-0.998 |
| Shape_SurfaceArea | 0.993 | 0.989-0.996 |
| Shape_Sphericity | 0.969 | 0.946-0.982 |
| Shape_Compactness | 0.977 | 0.960-0.987 |
| Shape_Roundness | 0.948 | 0.910-0.970 |
| Shape_Circularity | 0.971 | 0.950-0.983 |
| Shape_Longest1stAxis | 0.985 | 0.974-0.991 |
| Shape_Longest2ndAxis | 0.984 | 0.973-0.991 |
| Shape_PCA1stMajorSD | 0.998 | 0.997-0.999 |
| Shape_PCA2ndMajorSD | 0.998 | 0.997-0.999 |
| Shape_PCA3rdMajorSD | 0.995 | 0.991-0.997 |
| Fractal Feature |  |  |
| FractalDimension | 0.968 | 0.944-0.981 |

CT, computed tomography; SD, standard deviation; Histo, histogram; Min, minimum; Max, Maximum; Grad, gradient; GLCM, grey-level co-occurrence matrix; ASM, angular second moment; IDM, inverse different moment; Autocor, autocorrelation; CP, cluster prominence; CS, cluster shade; CT, cluster tendency; GLRLM,; grey-level run-length matrix; SRE, short run emphasis; LRE, long run emphasis; LGRE, low gray-level run emphasis; HGRE, high gray-level run emphasis; SRLGE, short run low gray-level emphasis; SRHGE, short run high gray-level emphasis; LRLGE, long run low gray-level emphasis; LRHGE, long run high gray-level emphasis; GNUN, gray-level non-uniformity normalized; RLNUN, run-length non-uniformity normalized; RP, run percentage; RV, run variance; RE, run entropy; PCA, principal component analysis
